# Improving activity and enantioselectivity of lipase via immobilization on macroporous resin for resolution of racemic 1- phenylethanol in non-aqueous medium

**DOI:** 10.1186/1472-6750-13-92

**Published:** 2013-10-29

**Authors:** Xiang Li, Shuangshuang Huang, Li Xu, Yunjun Yan

**Affiliations:** 1 Key Laboratory of Molecular Biophysics of the Ministry of Education, College of Life Science and Technology, Huazhong University of Science and Technology, Wuhan 430074, China

## Abstract

**Background:**

*Burkholderia cepacia* lipase (BCL) has been proved to be capable of resolution reactions. However, its free form usually exhibits low stability, bad resistance and no reusability, which restrict its further industrial applications. Therefore, it is of great importance to improve the catalytic performance of free lipase in non-aqueous medium.

**Results:**

In this work, macroporous resin NKA (MPR-NKA) was utilized as support for lipase immobilization. Racemic transesterification of 1-phenylethanol with vinyl acetate was chosen as model reaction. Compared with its free form, the enzyme activity and enantioselectivity (*ee*_s_) of the immobilized lipase have been significantly enhanced. The immobilized BCL exhibited a satisfactory thermostability over a wide range of temperature (from 10 to 65°C) and an excellent catalytic efficiency. After being used for more than 30 successive batches, the immobilized lipase still kept most of its activity. In comparison with other immobilized lipases, the immobilized BCL also exhibits better catalytic efficiency, which indicates a significant potential in industrial applications.

**Conclusion:**

The results of this study have proved that MPR-NKA was an excellent support for immobilization of lipase via the methods of N_2_ adsorption–desorption, scanning electron microscopy (SEM), energy dispersive spectroscopy (EDS) and Fourier transform-infrared spectroscopy (FT-IR). The improvement of enzyme activity and *ee*_s_ for the immobilized lipase was closely correlated with the alteration of its secondary structure. This information may contribute to a better understanding of the mechanism of immobilization and enzymatic biotransformation in non-aqueous medium.

## Background

In recent years, lipase (EC 3.1.1.3) has been widely applied in biotransformation reactions in aqueous and non-aqueous medium, because it can be used to catalyze hydrolysis and transesterification reactions, as well as synthesis of esters [1]. Especially, the ability of lipases to perform enantioselective biotransformation in preparation of pharmaceutical intermediates and chiral building blocks has made them increasingly attractive and promising [2]. Particular attention has been paid to the *Burkholderia cepacia* strain, which can produce versatile enzyme and be widely used for biodegradation, biological control and hydrolyzing biotransformation in various reactions [3]. The lipase from *Burkholderia cepacia* lipase (BCL) has high stability, alcohol tolerance and activity suitable for a broad spectrum of reactions substrates and media [4]. However, its free form usually exhibits low stability, bad resistance and no reusability, which restrict its further application in industry [5]. In most cases, these disadvantages of directly using free form lipase are common phenomena in other enzyme-catalyzed reactions [6,7]. Thus, the issue focusing on how to improve the catalytic properties of free lipase (such as activity, thermal stability and reusability) in non-aqueous medium is an important topic.

Immobilization has been proved to be one of the most useful strategies to improve catalytic properties of free enzyme [8]. There are several conventional immobilization approaches, such as adsorption, entrapment, encapsulation, and covalent binding [9,10]. Among them, adsorption is advantageous because of its procedural simplicity, low cost, high efficiency and ease of industrial application. Immobilized lipases via adsorption methods have been used in many reactions, such as ester synthesis, biodiesel production and enrichment of polyunsaturated fatty acids [5,11,12]. So far, various materials have been employed as supports for enzyme immobilization [13]. However, usage of MPRs in the resolution reaction has rarely been explored. Although many studies showed that immobilization could greatly enhance the catalytic performance of enzyme [14,15], till now, to the best of our knowledge, it is still unclear that why immobilization can enhance the activity and tolerance of lipases. Thus, it is important to elucidate the possible mechanism of this enhancement.

For this purpose, in this study, several methods (N_2_ adsorption–desorption, SEM, EDS and FT-IR) were employed to characterize the immobilized lipase in order to investigate probable mechanism for the enhancements of enzyme activity and enantioselectivity after immobilization. The enantioselective transesterification of racemic 1-phenylethanol with vinyl acetate was chosen as the model reaction so as to evaluate the enzyme activity/enantioselectivity (*ee*_s_) and to compare the catalytic efficiency between the free and immobilized lipases in non-aqueous medium [16], because secondary alcohols are often used as target substrates in lipase-catalyzed resolution reactions [17]. In addition, 1-phenylethanol is an essential building block and synthetic intermediate in many fields, such as fragrance in cosmetic industry, solvatochromic dye in chemical industries, ophthalmic preservative and inhibitor of cholesterol intestinal adsorption in pharmaceutical industries [18]. Moreover, numerous reports on transesterification of racemic 1-phenylethanol with vinyl acetate are available in the literature, we can easily compare the catalytic activity of immobilized BCL with other enzyme catalysts under similar reaction conditions.

Therefore, based on the above analysis, the main objectives of this work are: (1) to compare the properties of the free lipase and the immobilized lipase on MPRs based on the reaction parameters, such as temperature, water content, substrate molar ratio, and reaction time; (2) to investigate probable mechanism for the significant improvement of enzyme activity and enantioselectivity through various characterizations of the immobilized lipase; and (3) further to compare the catalytic efficiency between the immobilized BCL and other immobilized lipases.

## Results and discussion

### MPR selection

**Figure 1:**
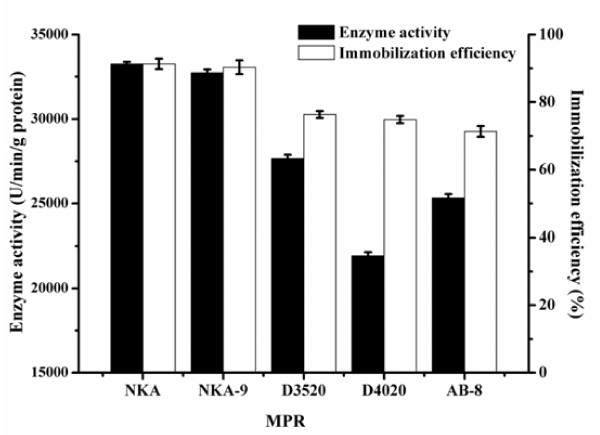
Effect of different kinds of MPRs on enzyme activity and immobilization efficiency.

The enzyme activities and immobilization efficiencies of the 5 types of MPRs are presented in Figure 1. The properties of MPRs, such as particle size, specific surface area and pore diameter, were listed in Table 1.

**Table 1:** Properties of MPRs used in the present study

**Items**	**Matrix structure**	**Density (g/mL)**	**Particle size (μm)**	**Specific surface area (m**^**2**^**/g)**	**Porosity (%)**	**Average pore diameter (nm)**
D3520	Crosslinked Polystyrene	1.02-1.06	300-1250	480-520	65-70	8.5-9.0
D4020	Crosslinked Polystyrene	1.08-1.12	300-1250	540-580	46-50	10-10.5
NKA	Crosslinked Polystyrene	1.02-1.06	300-500	570-590	78-80	20-22.0
AB-8	Crosslinked Polystyrene	1.02-1.08	480-520	480-520	42-46	13-14.0
NKA-9	Crosslinked Polystyrene	1.02-1.04	300-700	540-580	74-78	15.5-16.5

As shown in Table 1, MPRs were synthesized from inexpensive styrene. Actually, they had relatively low price ($ 5-12/kg). The result in Figure 1 showed that the enzyme activity and immobilization efficiency both were highest as compare with the other MPRs, when BCL was immobilized on MPR-NKA. The reason was mainly attributed to different specific surface area and pore diameter. Among these five types of MPRs, NKA had relatively higher specific surface area and average pore diameter (> 20 nm). Gao *et al*. [12] has also made similar conclusion that pore diameter of resin influences immobilization degree where immobilization degree increased with the increment of pore diameter. Therefore, MPR-NKA was chosen as the immobilization matrix in the following experiments.

### Effect of substrate molar ratio on enzyme activity/*ee*_s_ of the free and immobilized BCL

**Figure 2:**
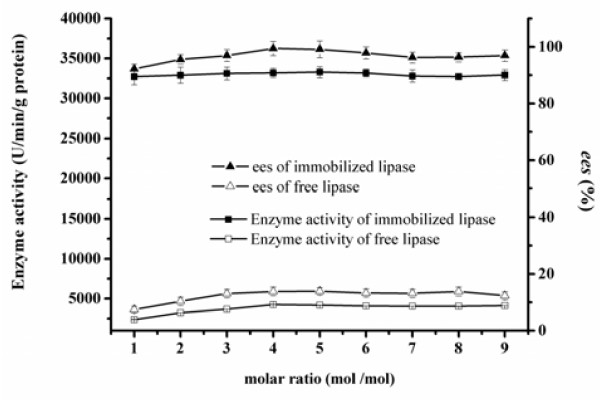
**Effect of substrate molar ratio on enzyme activity/ees of the free and immobilized BCL.** Reaction condition: 0.1 g free and BCL was added to 5 mL n-heptane containing 1 mmol 1-phenylethanol, 1-9 mmol vinyl acetate. The reactions were performed at 35°C, 200 rpm for 1 h. The data were measured three times using the same sample and means. Vertical bars represent standard deviation.

As shown in Figure 2, the effect of substrate molar ratios of vinyl acetate to racemic 1-phenylethanol from 1:1 to 10:1 has been investigated. It is generally believed that the acyl donor concentration would affect the reaction equilibrium, because the excess amount of vinyl acetate could drive the reversible reaction to the right side. For the free lipase, it could be observed that the highest enzyme activity and *ee*_s_ was obtained when the substrate ratio was 4:1. However, it could also be found from Figure 2 that further increase of substrate ratio had little effect on the enzyme activity and enantioselectivity of the free and immobilized BCLs when the molar ratios were more than 4:1.

### Effect of water content on enzyme activity/*ee*_s_ of the free and immobilized BCL

**Figure 3:**
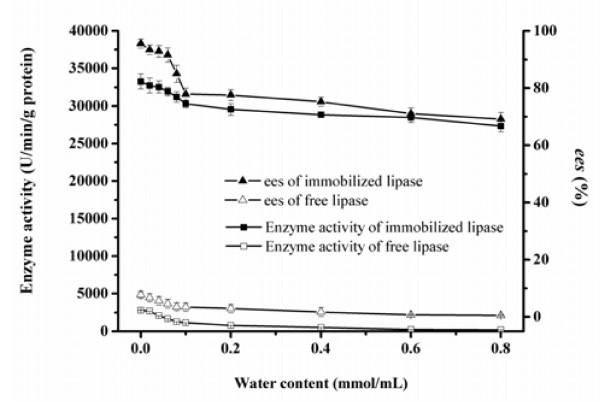
**Effect of water content on enzyme activity/ees of the free and immobilized BCL.** Reaction condition: 0.1 g BCL was added to 5 mL n-heptane containing 1 mmol 1-phenylethanol, 4 mmol vinyl acetate. The reactions were performed at 35°C, 200 rpm for 1 h. The data were measured three times using the same sample and means. Vertical bars represent standard deviation.

When a reaction is performed in organic medium, the enzyme activity would be affected by micro-water in the reaction system [19]. In addition, water has different effects on the enzyme activity and enantioselectivity in various lipase-catalyzed reactions [20]. The influence of water on the BCL-catalyzed reaction was investigated in a range of water contents from 0.02 mmol/mL to 0.80 mmol/mL. As can be seen in Figure 3, for the free lipase, the enzyme activity decreased significantly, while the *ee*_*s*_ was observed to be correlated well with the decrease of enzyme activity. It indicates that high water content may lead to an increase in hydrolysis, resulting in the decrease in transesterification activity of the enzyme. For the immobilized lipase, the enzyme activity and *ee*_*s*_ also showed a similar decrease in tendency, which can be explained as that extra water would accumulate inside the immobilized lipase and influence the flexibility of the protein [21]. Therefore, there is no necessity to add extra water during the reaction, as the immobilized lipase has contained necessary water to maintain its active conformation during immobilization process.

### Effect of temperature on enzyme activity/*ee*_s_ of the free and immobilized BCL

**Figure 4:**
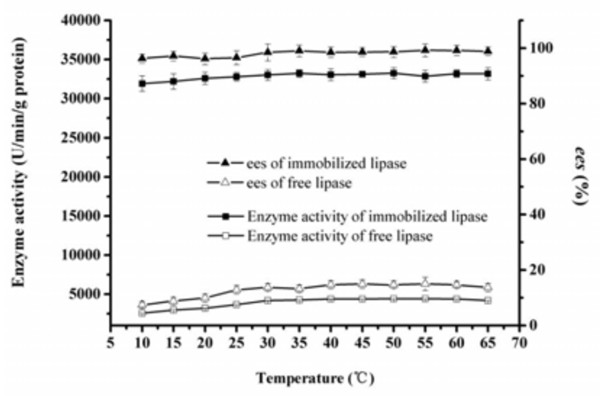
**Effect of temperature on enzyme activity/ ees of the free and immobilized BCL.** Reaction condition: 0.1 g BCL was added to 5 mL n-heptane containing 1 mmol 1-phenylethanol, 4 mmol vinyl acetate. The reactions were performed at different temperatures, 200 rpm for 1 h. The data were measured three times using the same sample and means. Vertical bars represent standard deviation.

The effect of temperature from 10 to 65°C on the enzyme activity and *ee*_*s*_ of the free and immobilized BCL for resolution of (*R*, *S*)-1-phenylethanol was examined (Figure 4). For the free lipase, the enzyme activity increased with the increment of temperature when temperature was below 55°C, which agrees with the observation of Phillips [22]. The *ee*_*s*_ also grew with the increase of temperature. When the temperature was above 55°C, both enzyme activity and *ee*_*s*_ decreased, which indicates that higher temperature would inhibit enzyme activity. For the immobilized lipase, enzyme activity and *ee*_*s*_ were a little lower when temperature was below 20°C, which was attributed to heterogeneous mixture of substrate, acyl donor and organic medium at lower temperature. Compared with the free lipase, enzyme activity and *ee*_*s*_ of the immobilized lipase exhibited no obvious decrease when temperature was over 20°C, which suggested immobilization could improve the thermostability of lipase.

### Effect of reaction time on conversion/*ee*_s_ of the free and immobilized BCL

**Figure 5:**
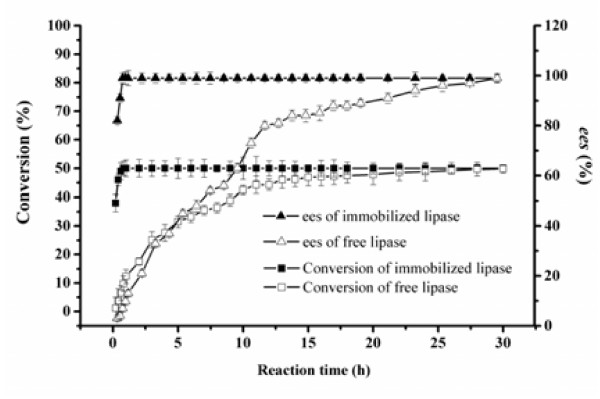
**Effect of reaction time on conversion/ees of the free and immobilized BCL.** Reaction condition: 0.1 g BCL was added to 5 mL n-heptane containing 1 mmol 1-phenylethanol, 4 mmol vinyl acetate. The reactions were performed at 35°C, 200 rpm for different reaction times. The data were measured three times using the same sample and means. Vertical bars represent standard deviation.

As shown in Figure 5, the conversion of the free lipase increased with the reaction time at a very slow rate, and *ee*_s_ showed the same tendency. The reaction reached equilibrium at a conversion near to 50%, while *ee*_s_ was close to 100%. It indicated that BCL had a good preference for (*R*)-1-phenylethanol, and all of (*R*)-1-phenylethanol had been nearly converted into (*R*)-phenylethyl acetate, while (*S*)-1-phenylethanol remained unchanged in the reaction solution. When the conversion and *ee*_s_ of the free lipase reached 50% and 99% at 30 h, its enzyme activity was only 924.1 U/min/g protein, while the corresponding *E* value was more than 200. On the contrary, the immobilized BCL showed a very high initial reaction rate, the reaction equilibrium (*ee*_s_ close to 99%, conversion near to 50%) could be achieved within 30 min; the corresponding enzyme activity was 33,266.7 U/min/g protein. The enzyme activity of the immobilized BCL was 36 folds enhancement over the free lipase powder.

### Operational stability and reusability of the immobilized BCL

**Figure 6:**
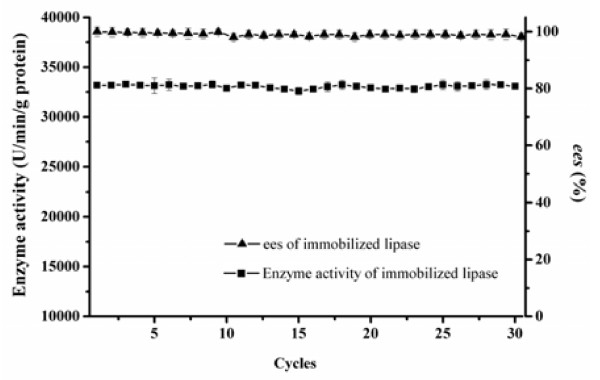
**Reusability of the immobilized BCL.** Reaction condition: 0.1 g BCL was added to 5 mL n-heptane containing 1 mmol 1-phenylethanol, 4 mmol vinyl acetate. The reactions were performed at 37°C, 200 rpm for 1 h. The data were measured three times using the same sample and means. Vertical bars represent standard deviation.

The reusability of the immobilized lipase is vital for cost-effective usage in the large-scale applications. In this study, the immobilized lipase can be easily separated from the reaction mixture by centrifugation. After every batch, the immobilized BCL was washed with n-heptane to remove traces of substrate and products. Then, it was ready to be used for the next batch reaction under the same conditions. As shown in Figure 6, there was nearly no loss in enzyme activity and *ee*_s_ after the immobilized BCL had been continuously used for at least 30 cycles. Hence, it has been very clear that the immobilized lipase exhibited an excellent reusability. Therefore, the immobilized BCL is applicable not only to the batch reaction, but also to the continuous reaction and different reactor instruments.

### The BJH pore size distributions of MPR-NKA

**Figure 7:**
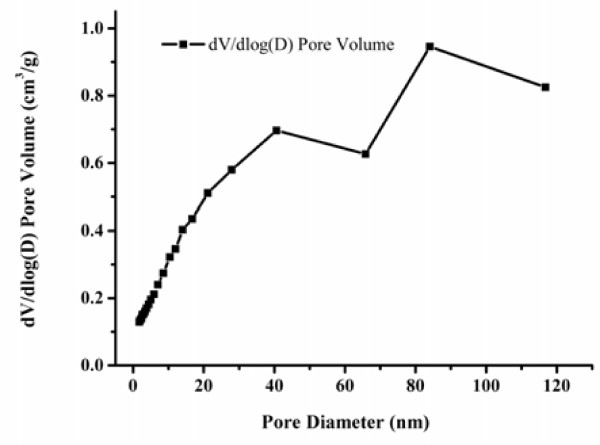
BJH pore size distributions.

Compared with pure MPR-NKA, MPR-NKA adsorbed with lipase showed a decrease in the pore volume (from 0.985 m^3^/g to 0.881 m^3^/g). As shown in Figure 7, MPR-NKA contained relatively large pore volume, contributing to a better adsorption of lipase during immobilization. Compared with the pore volume of pure MPR-NKA, the decrease in the pore volume was attributed to the occupation of the enzyme in the pore channels, which indicated that BCL had been immobilized on MPR-NKA. It has been reported that the pore diameter should be at least four- to five-fold the protein diameter in order to prevent restrictions to the access of the enzyme [23]. Lipases are macromolecules of protein, with molecular weights about 40,000–60,000 Da [24]. Moreover, the structure of *Pseudomonas cepacia* lipase has been resolved (rscb accession No. 1OIL) [25,26]. It was very easy to estimate the diameter of single BCL molecule (about 5 nm), so the minimum pore diameter should be at least 20 nm. As shown in Figure 7, the diameters of most pores were from 20 to 110 nm, which matched the requirement of pore diameter.

### SEM and EDS analysis

**Figure 8:**
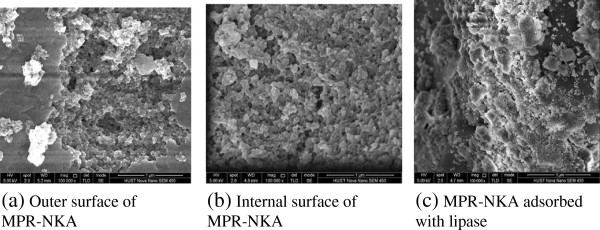
**SEM images of the pure MPR-NKA and MPR-NKA immobilized BCL. (a)** Outer surface of MPR-NKA (Magnification: 100 000, Accelerating Voltage: 5.00 kV); **(b)** Internal surface of MPR-NKA (Magnification: 100 000, Accelerating Voltage: 5.00 kV); **(c)** MPR-NKA adsorbed with lipase (Magnification: 100 000, AcceleratingVoltage: 5.00 kV).

**Figure 9:**
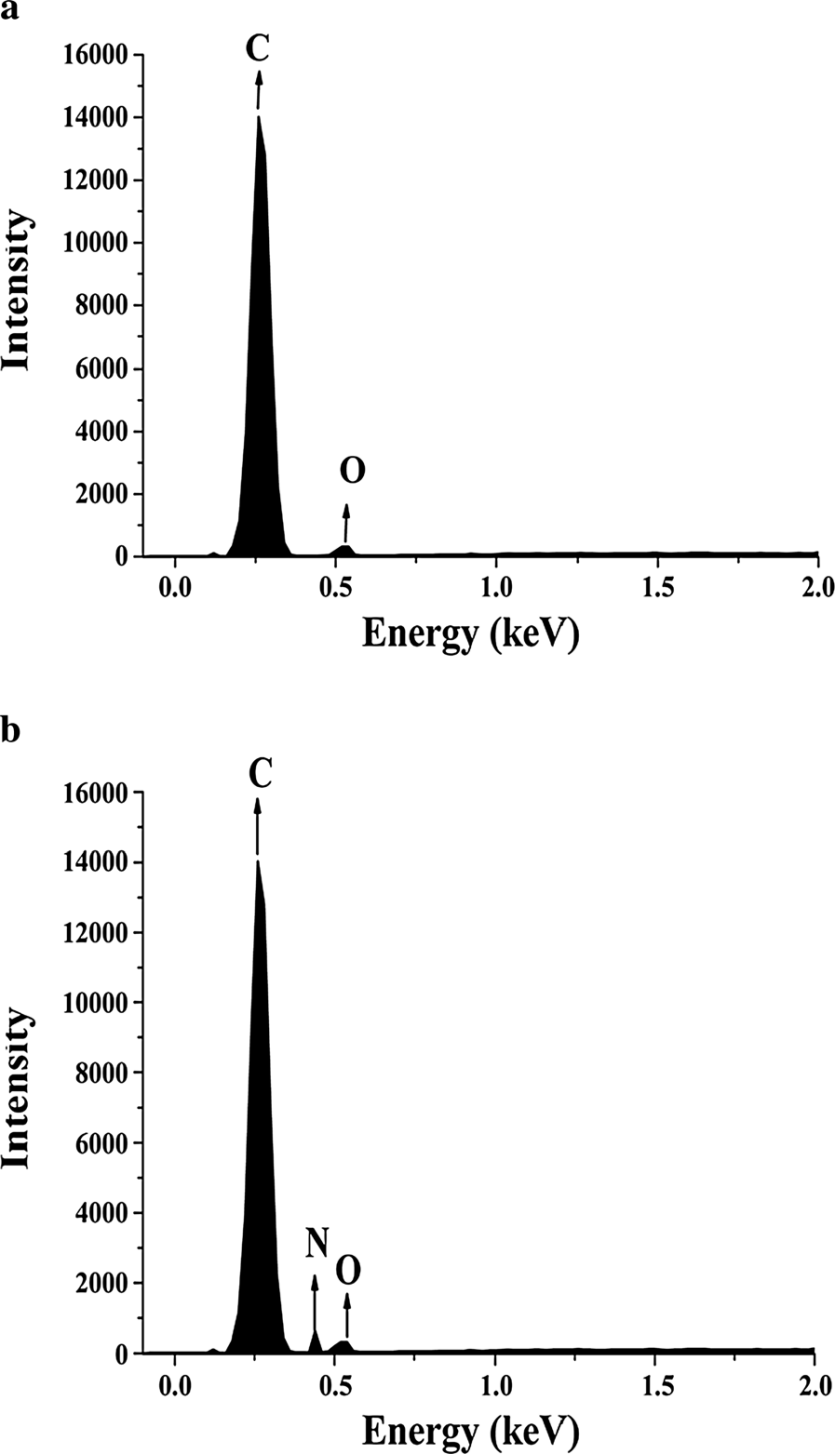
**EDS spectra of MPR-NKA and MPR-NKA immobilized BCL. (a)** EDS spectra of MPR-NKA; **(b)** EDS spectra of MPR-NKA adsorbed with lipase.

As shown in Figure 8, the detailed information about pore size distribution and shape of MPR-NKA has been given by SEM micrographs. Figure 8(a,b) show the surface and internal surface of MPR-NKA, respectively. It can be seen that MPR-NKA has various pore volumes on its surface and inside, which also proves the conclusion from BJH pore size distribution. Figure 8(c) showed the pore volumes of MPR-NKA adsorbed with lipase, which indicates that BCL has been immobilized on MPR-NKA. This can also be confirmed by EDS analysis. The result of EDS (in Figure 9a) displays that C and O are present without other elements in pure MPR-NKA (H element could not be detected in EDS). However, the elements of C, O and N are present after BCL immobilized on MPR-NKA in Figure 9b, which also proves that the immobilization of BCL on MPR-NKA was successful [27].

Moreover, some researchers have reported that the inner surface may not be fully utilized for lipase adsorption even if the pore size is big enough during the immobilization process [11,28]. As shown in the Figure 8 (a,b), the outer and internal surface of pure MPR-NKA were full of various pores before adsorption. After immobilization, surface of MPR-NKA was covered by lipase, and pores on the surface of MPR-NKA could not be found (in Figure 8c), which indicates that the lipase has almost been adsorbed by the MPR-NKA. It meant that the internal surface of MPR-NKA had been fully utilized, which was the possible reason for the high thermostability, organic solvent tolerance and operational stability of BCL immobilized on MPR-NKA.

### Secondary structure analysis of the free and immobilized BCL by FT-IR spectroscopy

**Figure 10:**
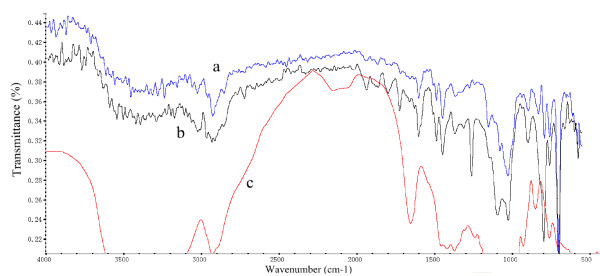
FT-IR spectra of (a: blue line) the pure MPR-NKA; (b: black line) MPR-NKA immobilized BCL and (c: red line) free BCL.

As known, protein has strong absorbance spectrum in the amide I region (1700–1600 cm^−1^) mainly due to the C = O bending vibration [29]. The amide I band of proteins contains component bands that represent different secondary structure elements such as the α-helix, β-sheet, β-turn and random coil. Their main absorbance spectra were: α-helix: 1650–1658 cm^−1^, β-sheet: 1620–1640 cm^−1^, β-turn: 1670–1695 cm^−1^, and random coil: 1640–1650 cm^−1^, respectively [30]. FT-IR spectra of the pure MPR-NKA (Figure 10a); BCL immobilized MPR-NKA (Figure 10b) and free BCL (Figure 10c) were shown in Figure 10, respectively. Compared with spectra of pure MPR-NKA, BCL immobilized MPR-NKA had a characteristic peak at 1700–1600 cm^−1^, which could also be observed in the spectra of free BCL.

As shown in Table 2, the secondary structure element content of free lipase was: α-helix: 28.3%, β-sheet: 21.4%, β-turn: 25.8%, and random coil: 24.5%, respectively. After immobilization, the immobilized BCL showed a decrease in α-helix (11.8%) and β-turn (15.9%); an increase in β-sheet (42.6%) and random coil (29.8%). Foresti *et al*. reported that interfacial activation had been found when the lipase was adsorbed onto hydrophobic supports. The immobilized lipase was fixed in an open conformation and enhanced enzymatic activity was achieved [31]. Gao *et al*. pointed out that lipases are interfacial-active enzymes with lipophilic domains and can adopt both open and close conformations. The ionic microenvironment around lipase molecule, which was formed during immobilization procedure in buffer solution at a certain pH value, could be maintained as employed in organic solvent. This is so-called “pH memory effect”, which helps to induce conformational changes of lipase resulting in the active form. Therefore, this would allow free access of the substrate to the active site of the immobilized lipase and increase activity of the immobilized lipase [12].

**Table 2:** Quantitative estimation of the secondary structure elements of free and immobilized BCL

**Solvent**	**α- Helix (%)**	**β- Sheet (%)**	**β- Turn (%)**	**Random coil (%)**
Free lipase	28.3 ± 1.09	21.4 ± 2.21	25.8 ± 0.91	24.5 ± 0.21
Immobilized lipase	11.8 ± 3.21	42.6 ± 1.21	15.9 ± 0.81	29.8 ± 0.71

### Comparison with other immobilized lipases

Compared with other immobilized lipases, the immobilized BCL exhibited a much higher catalytic efficiency. Chua *et al*. reported that immobilized lipase ChiroCLEC-PC (cross-linked enzyme crystals of *Pseudomonas cepacia* lipase) was used for the resolution of racemic 1-phenylethanol in organic solvents (including heptane) with different log *P* values, while the maximal initial rate of reaction was 473.5 ± 10 μmol/min and the reaction reached equilibrium conversion at 45% after 100 mins of reaction [18]. Compared with the cross-linked enzyme crystals method, the immobilized BCL showed a better catalytic efficiency (based on initial reaction rate and final conversion value). Wang *et al*. reported that lipase from *B. cepacia* was encapsulated inside zirconia particles by biomimetic mineralization of K_2_ZrF_6_. After 48 h reaction under the optimal conditions, their immobilized lipase reached 49.9% with higher *ee*_s_ of 99.9%, however, after 6 cycles, the conversion and *ee*_s_ were only 43% and 85%, respectively [32]. Compared with the approach of encapsulating lipase within zirconia induced by protamine, our immobilized BCL exhibited a better reusability in the successive batch experiments.

In order to compare the catalytic efficiency between our immobilized BCL and several commercially available immobilized lipases usually used in literature, the *ee*_*s*_ and conversions of Novozyme 435, Lipozyme RM IM, and Lipozyme TL IM were measured respectively. Under the same conditions of substrate molar ratios (vinyl acetate to racemic 1-phenylethanol) 4:1; reaction time 0.5 h, reaction temperature 35°C, 0.1 g immobilized lipase and 5 mL solvent (heptane), their *ee*_*s*_ was 75%, 24% and 15%, respectively. The corresponding conversions were 43.3%, 2.6% and 4.8%, respectively. It can be seen that our immobilized BCL (*ee*_*s*_ 99%; conversion 49%) is much better than the commercially avaialable immobilized lipases in catalyzing enantioselective transesterification of 1-phenylethanol with vinyl acetate.

## Conclusion

In this study, results were significantly enhanced in terms of enzyme activity and *ee*_s_ when BCL immobilized on MPR-NKA. Compared with the free BCL, the immobilized BCL had better thermostability and excellent reusability in non-aqueous medium. Combined strategies (N_2_ adsorption–desorption, SEM and EDS) were used to characterize the immobilized lipase, which proved that MPR-NKA was an excellent support for lipase immobilization. FT-IR analysis also indicated that improvement of enzyme activity and *ee*_s_ was closely correlated with the alteration of its secondary structure. Compared with the other immobilized lipases, the immobilized BCL exhibits a better catalytic efficiency, indicating a great potential for industrial applications.

## Methods

### Materials

Lipase from *B. cepacia* was purchased from Amano Enzyme Inc. (Nagoya, Japan). Racemic and optically pure 1-phenylethanol was got from Alfa Aesar Co., Ltd (P. R. China). 1-Phenylethyl acetate; (*R*)- and (*S*)- 1-phenylethanol were bought from Sigma-Aldrich Co., Ltd (St. Louis, Missouri, USA). Novozym 435 (from *Candida antarctica*), Lipozyme RM IM (from *Rhizomucor miehei*) and Lipozyme TL IM (from *Thermomyces lanuginose*) were purchased from Sigma-Aldrich Co., Ltd (St. Louis, Missouri, USA). MPR was product of Tianjin Nankai Hecheng S & T Co., Ltd (China). All organic solvents and other reagents were of analytical grade and were obtained commercially from Sinopharm Chemical Reagent Co., Ltd (Shanghai, China).

### Preparation of immobilized lipase

The procedures of immobilization were described as follows: 1 g MPR and 5 mL 99% ethanol was added into a 25 mL tube, then the mixture was put in 30°C shaking incubator at 200 rpm for 2 h to wash out the residual catalyst and impurities. The ethanol was removed after MPR precipitated to the bottom of the tube. The residual MPR was washed with distilled water for three times. 5 mL 0.05 M phosphate buffer (pH 7) was mixed with the residual MPR, the mixture was kept for 12 h at 30°C. Then, the buffer was removed. After this pretreatment, MPR was kept in the tube. 0.8 g free BCL powder was dissolved in the 5 mL 0.05 M phosphate buffer (pH 7), this solution was loaded into the tube to mix with the MPR. The tube was stirred in a rotary shaker with a speed of 200 rpm at 30°C for 2 h. The suspension was separated after MPR precipitated to the bottom of the tube. The immobilized BCL (MPR adsorbing the lipase) was washed with 5 mL 0.05 M phosphate buffer (pH 7) for three times to remove the unadsorbed lipase in the surface of the MPR, and then, protein content of the lipase solution and washed water was determined by the method of Bradford [33]. Five types of MPRs were used so as to choose the best immobilization support.

### Lipase activity and protein content measurements

The enzyme activity was determined using 1-phenylethanol and vinyl acetate as substrate. One unit (U) of the enzyme activity was defined as the amount of the enzyme which produces 1 μmol α -phenylethyl acetate per minute under the assay conditions. The reactions were performed in a 50 mL stoppered flask at 35°C and 200 rpm for 1 h. The assay conditions were used except when otherwise stated in the text. The protein content of free and immobilized BCL was 0.58 wt% and 0.50 wt%, respectively. Immobilization efficiency (%) was estimated as Eq. 1.

(1) $$\begin{array}{c} \mathrm{Immobilization}\;\mathrm{efficiency}\;\left(\%\right)\\ \;=\frac{\mathrm{immobilized}\;\mathrm{protein}}{\;\mathrm{total}\;\mathrm{loading}\;\mathrm{protein}}\times 100\% \end{array}$$

### Reaction procedure

Before usage, the organic solvent was dried over 4 Å molecular sieves. Under the above mentioned conditions, reactions were carried out in 5 mL pure heptane, containing 1 mmol racemic 1-phenylethanol, 4 mmol vinyl acetate and 0.1 g free or immobilized BCL. The reaction mixture was put in a 50 mL stoppered flask at 35°C and 200 rpm for 1 h. These conditions were used except when the reaction parameters (molar ratio; temperature; reaction time) needed to be changed in the following text. The above experiments were all conducted in triplicate. After the reactions, the free or immobilized lipase was removed by centrifugation. Then, the samples were filtered through a 0.44 μm filter and analyzed by HPLC.

### Analysis and calculation

The samples were analyzed by HPLC (Model 2300–525 SSI. Co., Ltd USA) using a Chiralcel OD-H column (4.6 mm × 250 mm, Daicel Chemical, Japan). Samples (5 μL) were eluted by a mixture of n-hexane: 2-propanol (95:5, v/v) at a rate of 1.0 mL/min, and detected at a wavelength of 254 nm (Model 525 UV Detector SSI. Co., Ltd USA). The retention time of (*R*)- and (*S*)-1-phenylethanol in the Chiralcel OD-H column was 7.28 and 8.23 min, respectively.

According to method described by Chen *et al.*[34], enantioselectivity was expressed as *E* value and calculated by Eq. 2, *ee*_*s*_ by Eq. 3, and *C* by Eq. 4.

(2) $$E=\frac{\ln\left[\left(1-C\right)\left(1-\mathrm{ees}\right)\right]}{\ln\left[\left(1-C\right)\left(1+\mathrm{ees}\right)\right]}$$

(3) $$\mathrm{ees}=\frac{S-R}{S+R}$$

(4) $$C=\frac{S0+R0-\left(S+R\right)}{S0+R0}$$

where, *C* represents the substrate conversion, *ee*_*s*_ stands for the substrate enantiomeric excess, *S*_0_ and *R*_0_ respectively represent the concentrations of the (*S*)- and (*R*)-enantiomers of 1-phenylethanol before reaction, *S* and *R* are the concentrations of the (*S*)- and (*R*)-enantiomers of 1-phenylethanol after reaction.

### Characterization of the immobilization support with N_2_ adsorption–desorption

The specific surface area, pore volumes, and average pore diameters were measured by nitrogen adsorption–desorption equipment (ASAP 2020 V4.00, Micromeritics Instrument Ltd, Shanghai). The specific areas of the MPR-NKA were calculated by the Brunauer–Emmett–Teller (BET) method, and the distributions of pore diameters were estimated by the desorption branches of the isotherms with the Barrett–Joyner–Halenda (BJH) model.

### Characterization of the immobilized BCL by SEM and EDS

The immobilized BCL was analyzed with SEM and EDS (Nova Nano SEM 450, FEI Company, Eindhoven, Netherlands). The samples were coated with gold using a sputter coating system and measured at an acceleration voltage of 5 kV.

### FT-IR spectroscopy

The samples were mixed with KBr and pressed into pellets. FT-IR measurements in the region of 400-4000 cm^-1^ were recorded at 25°C by Vextex 70 FT-IR spectrometer (Bruker, Germany) with the nitrogen-cooled, mercury–cadmium– tellurium (MCT) detector. The spectrum acquisition (all samples were overlaid on a zinc selenide ATR accessory) is from IR spectra. The infrared spectrum of KBr has been subtracted from the infrared spectrum during each measurement. The conditions of the measurements were as follows: 20 kHz scan speed, 4 cm^-1^ spectral resolution, 128 scan co-additions, and triangular apodization. The secondary structure element content was estimated by software PeakFit version 4.12 according to the method described by Yang *et al. *[35]*.*

## Competing interests

The authors declare that there are no competing interests.

## Authors’ contributions

XL(i) designed the study, carried out the experiments and drafted the manuscript. SH contributed to the measurement of protein content and SEM. LX(u) revised the language. YY supervised the work and finalized the manuscript. All authors have read and approved the final manuscript.
